# Low Plasma Lipids Are Associated with Relapsing and Lethal Visceral Leishmaniasis in HIV-Infected Patients

**DOI:** 10.3390/pathogens13060450

**Published:** 2024-05-25

**Authors:** Renata V. S. Silva, Silvia R. B. Uliana, Jenicer K. U. Y. Yasunaka, Cláudio S. Veloso, Emille Sousa, Maria M. L. Ferreira, Vivianne S. Carvalho, Gabriel R. Ferreira, Dorcas L. Costa, Carlos H. N. Costa

**Affiliations:** 1 Laboratório de Leishmanioses, Departamento de Medicina Comunitária, Universidade Federal do Piauí, Teresina 64002-510, PI, Brazil; renatisvieira@gmail.com; 2 Laboratório de Leishmanioses, Instituto de Ciências Biomédicas, Universidade de São Paulo, São Paulo 05508-090, SP, Brazil; uliana.silvia@gmail.com (S.R.B.U.); jenicerk@usp.br (J.K.U.Y.Y.); 3 Instituto de Doenças Tropicais “Natan Portella”, Teresina 64002-510, PI, Brazil; csveloso@outlook.com (C.S.V.); dorcas.lc@gmail.com (D.L.C.); 4 Laboratório de Leishmanioses, Centro de Inteligência em Agravos Emergentes e Negligenciados, Teresina 64002-510, PI, Brazil; emilleasousa@gmail.com (E.S.); michellefl13@hotmail.com (M.M.L.F.); 5 Centro de Diagnóstico, “Dr. Raul Bacellar”, Fundação Municipal de Saúde, Teresina 64600-000, PI, Brazil; viviannecarvalhomg@gmail.com; 6 Department of Microbiology-Infectious Disease and Immunology, Faculty of Medicine, University Laval, Quebec, QC G1V 0A6, Canada; ferreira.rgabriel@gmail.com; 7 Departamento de Mãe e Filho, Universidade Federal do Piauí, Teresina 64002-510, PI, Brazil; 8 Departamento de Medicina Comunitária, Universidade Federal do Piauí, Teresina 64002-510, PI, Brazil; 9 Laboratório de Pesquisas em Leishmanioses, Centro de Investigações em Agravos Tropicais Emergentes e Negligenciados, Instituto de Doenças Tropicais “Natan Portella”, Universidade Federal do Piauí, Rua Artur de Vasconcelos 151, Teresina 64002-510, PI, Brazil

**Keywords:** visceral leishmaniasis, *Leishmania infantum*, HIV/AIDS, lipid profile, cachexia

## Abstract

Visceral leishmaniasis (VL) results from protozoa *Leishmania infantum* and *L. donovani* infection. This study investigated whether host factors would explain the relapses. First, susceptibility to amphotericin B of *L. infantum* isolates was evaluated in vitro. Then, clinical data and the lipid profile of patients with relapsing and non-relapsing VL were assessed. Susceptibility to amphotericin B was similar between the isolates. CD4+ lymphocytes were reduced in both groups of patients in the first episode and with relapsing VL. Still, the strongest blood cell indicator associated with relapses was low total lymphocyte counts. Total plasma cholesterol, high-density lipoprotein, low-density lipoprotein, and, uniquely, triglycerides of the six individuals in the first episode and twenty-three with relapsing VL were lower in relapsing patients than those in the first episode. Deceased patients had extremely low low-density lipoprotein. After CD4+ decreases, lymphocyte CD8+ reduction is the final stage of immunological failure. The lower lipid concentrations appear to be secondary to the depletion of fat stores by inflammation-induced cachexia and fat exhaustion provoked by the co-occurrence of both diseases, which can finally lead to death.

## 1. Introduction

Visceral leishmaniasis (VL), or kala-azar, is a vector-borne disease caused by the intracellular parasites *Leishmania infantum* and *L. donovani*, transmitted by sandflies. VL is characterized by prolonged fever, pallor, wasting, hepatosplenomegaly, and pancytopenia [1]. A protracted systemic inflammation mediated by cytokines is triggered [2] and fueled by the liver acute-phase response (APR) generated by the action of IL-6 [3]. The disease kills around 10% of patients [4]. Among those cured, some have relapses and require further treatments [5].

VL became a significant opportunistic disease of AIDS, where coinfected patients maintain low CD4+ counts. According to international guidelines, these patients are treated with a longer course of liposomal amphotericin B followed by secondary prophylaxis every two to three weeks. The response is poorer than in HIV-uninfected patients, even in cases where adherence to antiretroviral therapy (HAART) and with undetected viral load [6]. However, only a few clinical trials have been conducted on the efficacy of drug therapy for the treatment of this immunosuppressed population [7]. VL relapse is a new episode of VL that occurs after the initial treatment and is frequent in patients with AIDS. Individuals with VL and AIDS represent a challenge in clinical management because they are chronically symptomatic and have higher rates of drug toxicity, relapses, and mortality [8].

Although unusual, multiple relapses can be due to parasite resistance to amphotericin B [9]. Low drug exposure caused by inadequate dosing can explain treatment or secondary prophylaxis failure [10]. However, host factors may be responsible for therapeutic failures, such as low CD4+ cell counts before treatment, without displaying drug resistance. Lipid metabolism is another possible reason since amphotericin B is associated with lipoproteins. Additionally, total cholesterol (TC) and lipoprotein levels are significantly altered in patients with VL and AIDS, and cholesterol-rich diets modify drug concentration, toxicity, and tissue distribution of amphotericin B [11].

Therefore, as an attempt to understand the factors linked to relapses and disease severity of patients with VL coinfected with HIV, this study verifies the sensitivity to amphotericin B of *L. infantum* isolated from HIV-infected patients with and without relapses to rule out the possibility of drug resistance to *L. infantum* and then investigates the association of the plasma lipid profile and other host factors with relapses.

## 2. Methods

Two groups of VL patients with AIDS were retrospectively studied. One group consisted of five patients for testing amphotericin B sensitivity. Frozen stored isolates of *L. infantum* from four patients in their second VL episode and one from the patients in the first VL episode were tested for drug sensitivity. The other group was composed by of 29 patients divided into two subgroups. Twenty-three had relapsing VL and six were in the first episode of the disease; their medical records and lipid profiles were analyzed. All were hospitalized at a reference hospital in two subgroups in Teresina, Brazil, where they were treated for VL with liposomal amphotericin B at a dose of 3 mg/kg daily for two weeks. Besides treatment, patients received secondary prophylaxis for VL with liposomal amphotericin B at a dose of 3 mg/kg biweekly and HAART. The available patient information spans May 2014 to August 2020. All patients with reagent serology for HIV with clinical signs of VL and with a diagnosis of VL confirmed by parasitological methods were included.

*L. infantum* isolates were thawed and transferred to Novy, MacNeal, and Nicolle (NNN) medium with Schneider’s medium supplemented with heat-inactivated sterile fetal bovine serum (Gibco^®^), penicillin, and human urine. The samples were transferred and kept in a BOD incubator at 26 °C. For the in vitro assay with amphotericin B, M199 medium (Sigma-Aldrich, St. Louis, MO, USA) at 25 °C, supplemented with 10% heat-inactivated fetal calf serum (FCS) and 0.25% hemin, was used. Amphotericin B and 3-(4,5-dimethyl thiazol-2-yl)-2,5 diphenyl tetrazolium bromide (MTT) were acquired from Sigma-Aldrich (St. Louis, MO, USA). The MTT test was performed according to Zauli-Nascimento et al. [12]. All experiments included a reference strain of *L. infantum* (MHOM/BR/2005/NLC) as a control. The lipid levels were compared to the cumulative number of liposomal amphotericin B doses used. For this analysis, a 50 mg vial of the drug was the unit of analysis.

Lipid fractions were analyzed from plasma samples stored at −20 °C. They were processed with the Incca Bit Analisa equipment (Belo Horizonte, Brazil). The Analisa reagent kit (Belo Horizonte, Brazil) for TC, triglycerides (TG), and high-density lipoprotein (HDL) was used. Low-density lipoprotein (LDL) and very low-density lipoprotein (VLDL) values were obtained from Friedewald’s formula (VLDL = TG/5; LDL = TC − HDL − VLDL).

For data analysis of the in vitro assay*,* the EC50 values were expressed as mean and standard error (SEM), produced by GraphPad Prism 7.04 software. For human data analysis, the usual procedures of descriptive statistics were used. The Shapiro–Wilk test was used to check whether the data followed a normal distribution. For continuous and category variables, the Student’s *t*-test and Fisher’s exact test were performed. Pearson’s and Spearman’s correlation tests were used according to the data distribution. Data were tabulated and analyzed with the software Stata™, version 15.1, College Station, TX, USA.

## 3. Results

Among the five patients from whom parasites were available to analyze the sensitivity to amphotericin B, three were males and two females, aged 38 to 55. Four isolates were taken from patients during a relapsing episode of VL, and one was isolated from a patient during the initial symptomatic phase. All had AIDS. The four patients with relapsing VL were on secondary amphotericin B prophylaxis, each one with two previous relapses.

The amphotericin B EC50 values for promastigotes of the five isolates obtained is shown in Table 1. All were below the EC50 of the reference isolate MHOM/BR/2005/NLC (0.051 µM). The minimum value was 0.024 µM, and the maximum was 0.042 µM. No significant differences were observed between the mean EC50 of the isolates.

Next, the lipid profile of the six patients in the first episode of VL and the twenty-three under ongoing secondary prophylaxis was evaluated. Their general characteristics are shown in Table 2. Most were men over 40 years old. Treatment for VL was performed with liposomal amphotericin B. However, two had been previously treated with amphotericin B deoxycholate. The most frequent clinical manifestations were weight loss, pallor, fever, and hepatosplenomegaly. Clinical manifestations of relapsing and non-relapsing episodes observed at admission were similar, except for the fever, which was less common in relapsing episodes (*p*-value = 0.046). Complete adherence to HAART was observed in 21/29 (75.9%), all with relapses. All patients who died had relapses (13.8%, *p*-value = 0.55). Death occurred from 9 to 69 months after the samples were collected.

Table 3 shows the laboratory data of those six patients in the first episode of VL and of those twenty-three with a relapsing progression. Among the total 29 participants, 23 (95.8%) had low hemoglobin. The leukocyte count was low in 20 participants (74.1%), and, like neutrophils, they were lower among those with relapses (*p*-value = 0.025 and 0.076, respectively). Relapsing patients had much lower lymphocytes (660/mm^3^ vs. 1664/mm^3^, *p*-value = 0.005). Thrombocytopenia was seen in 12 individuals (44.5%). There was marked hypoalbuminemia and hyperglobulinemia. CD4+ cell count was low in all patients and was not statistically distinct between relapses and the first episode (142 cells/mm^3^ vs. 152 cells/mm^3^), varying from 5 cells/mm^3^ to 406 cells/mm^3^. Only three patients had a CD4+ count above 200 cells/mm^3^; one was in the first episode, and two had a relapsing course. Mean CD8+ cells were lower in relapsing patients (606 cells/mm^3^) than in those in the first VL episode (955 cells/mm^3^), but the difference was not statistically significant (*p*-value = 0.222). Total lymphocytes were highly correlated with CD8+ (r = 0.69, *p*-value = 0.003) but not with CD4+ cells. All patients in the first episode had detectable viral load.

The Supplemental Table S1 shows the low concentrations of all types of lipids evaluated. The two groups of participants had mean TC much lower than the suggested threshold of 190 mg/dL, and all showed TG values below the recommended level. LDL was also much inferior to the 130 mg/dL cut-off in all patients. Twenty (68.9%) participants had HDL values below the 40 mg/dL threshold. There was no correlation between lipid levels and the cumulative number of liposomal amphotericin B doses used.

**Figure 1:**
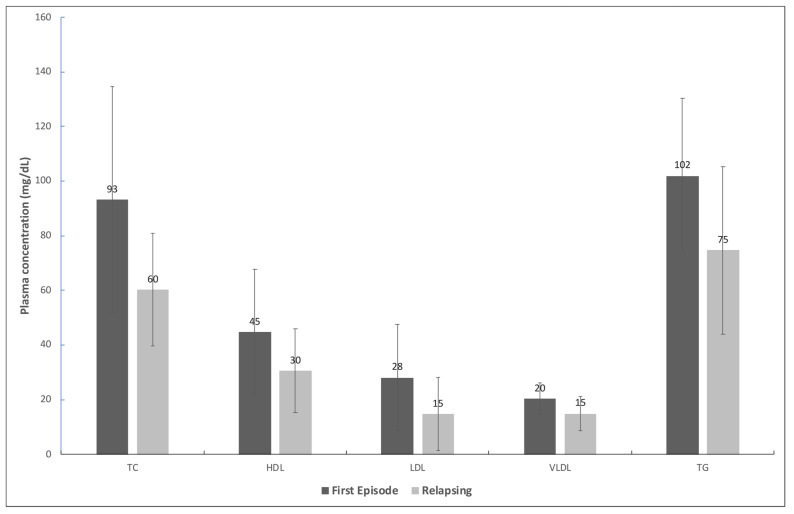
Mean plasma concentration and standard deviation of total cholesterol (TC), high-density lipoprotein (HDL), low-density lipoprotein (LDL), very low-density lipoprotein (VLDL), and triglycerides (TG) of patients with HIV in the first episode of and with a relapsing course of visceral leishmaniasis. All differences were statistically significant (*p*-value < 0.05).

The lipid profiles of the individuals with AIDS who had VL with a relapsing course and those in their first episode are shown in Figure 1. Total cholesterol was 93.2 mg/dL in the first episode and 60.2 mg/dL during a relapse (*p*-value = 0.005). The other lipids were also lower during relapses: HDL (44.7 vs. 30.5), LDL (28.1 vs. 14.8), VLDL (20.4 vs. 14.9), and TG (102.0 vs. 73.2), all with *p*-value < 0.05.

The lipid profile according to mortality is shown in Table 4. Impressively, the individuals who died had much lower LDL values than the survivors (3.6 mg/dL vs. 20.4 mg/dL, *p* = 0.038). There were two women and two men, in the range of 41 to 59 years of age; two underwent splenectomy as a salvage approach. The two not splenectomized died of *Pneumocystis jirovecii* pneumonia, and the two splenectomized died apparently of sepsis and portal thrombosis. The two with available HIV load measurements had 2.8 × 10^5^ viral RNA copies/mL and the other 4.4 × 10^3^ copies/mL. Their LDL levels were 0.4 mg/dL, 1.4 mg/dL, 2.0 mg/dL, and 10.4 mg/dL. No statistically significant associations with death were observed regarding TC, TG, and any other blood test.

## 4. Discussion

Amphotericin B is an essential drug for treating VL due to the development of drug resistance to other medications. However, in vitro resistance to amphotericin B is unusual [13]. Yet, the rescue of amphotericin B treatment with pentavalent antimonial has been previously described [14]. Here, the in vitro assay did not detect in vitro resistance, confirming previous findings [15]. The susceptibility assay was performed against promastigotes of the different isolates since it has been shown that there is a strong correlation of drug susceptibility between promastigotes and amastigotes of a given strain of *Leishmania* [16]. Moreover, the EC50 values of isolates of *L. infantum* obtained prior to and after treatment with amphotericin B were similar [17]. Therefore, the present findings indicated that treatment failure observed in the patients with relapsing VL and AIDS seems not to be due to parasite drug resistance.

The study population, comprising adult individuals with VL and AIDS, reflects the overall characteristics of preceding studies [18] but the observed mortality was higher than the mortality described for non-HIV-infected patients [19]. The most frequent clinical manifestations were similar to those of patients without HIV. However, fever was absent in nearly half of those with a relapsing course but present in all in the first episode of VL. Therefore, the fever mediators IL-1b, TNF-α, and IL-6 seem not to play the same intense role in the pathogenesis of the relapsing course as in the first episode. Accordingly, it has been shown that IL-6-driven C-reactive protein, a marker of the APR, is very high during the first episode of VL [20]. Hypoalbuminemia and hyperglobulinemia, also markers of APR, were impressively low and high, respectively, in a patient in the first episode but not so much in patients with a relapsing evolution.

Lower mean lymphocyte CD4+ count is the primary marker of VL relapse among AIDS patients [6]. However, in this study, the distinction of relapsing patients from patients in the first episode was not through the reduction in CD4+ cells but the CD8+ reduction, since CD4+ cells were already low in both groups [20,21]. Indeed, reductions in CD8+ cells have been noticed even in VL patients not coinfected with HIV [22]. Accordingly, as in the first episode of VL CD4+ count was already low, the data suggest that CD4+ depletion antecedes CD8+ loss, denoting a two-step process in which CD4+ reduction is initially dependent on HIV infection, allowing *L. infantum* proliferation and worsening inflammation and finally leading to CD8+ loss and reduced function, increasing immunosuppression, and causing future relapses. The mechanisms for total lymphocyte depletion in coinfected patients seem to involve continued systemic inflammation, thymic involution, reduced thymic output, chronic immune activation, increased lymphocyte apoptosis, T-cell exhaustion, and other subtle mechanisms [20,23]. In any case, recombinant IL-7, a growth factor for progenitor lymphoid cells, might be tried to rescue relapsing VL patients with AIDS as has been tried for patients with sepsis [24]. Important developments on the molecular mechanisms and networks of *Leishmania* and HIV confection have been recently and extensively reviewed, including the changes in the production/activities of the molecules IL-4, IL-12, IL-15, IFN-gamma, TGF-beta, RANTES, and MIP-1alpha, which have been shown to regulate the survival, activation, expansion, and activity of T CD8+ cells [25].

Since liposomal amphotericin B is the formulation of choice for the treatment and secondary prophylaxis of VL in patients with AIDS and interacts with serum lipoproteins in vitro and in the rabbit model, influencing the drug tissue concentration [11], the hypothesis that human lipids might be involved with liposomal amphotericin B activity against *Leishmania* was investigated. All measured lipids were low in patients with AIDS and VL, regardless of whether they relapsed [26]. This profile is distinct from those of patients with VL or AIDS, taken separately. In VL only, TC, HDL, and LDL are low. At the same time, VLDL and TG are higher than those of the controls or the recommended cut-offs [27]. The same profile is seen in patients with HIV infection and with AIDS not under HAART [28]. After the arrival of HAART, this profile changed according to the effects of the antiretroviral drugs [29]. Therefore, TG and, at a certain point, VLDL highlight a distinct lipid metabolism of patients with VL and AIDS together, in contrast to those with only one of the two conditions. The association between TC, LDL, HDL, VLDL, and TG with relapsing evolution suggests that the lipid profile should be investigated as a biomarker of VL relapse in cohort studies.

Two other critical points are common to VL and AIDS: inflammation and cachexia. Indeed, patients with VL have elevated markers of APR [3], as do patients with HIV or AIDS [30]. VL leads to cachexia [31] as does AIDS [32] and, especially, both diseases simultaneously [20]. Patients with APR, like in sepsis and trauma, also have low TC, HDL, and LDL, usually with high TG [33,34]. Protein–energy malnutrition shows a trend for low TG and other lipids as observed in VL patients with AIDS: low TC, HDL, LDL, and also low TG [35,36,37]. This picture suggests that a possible mechanism for the lipid profile seen in VL and AIDS, with all measured lipids at a lower concentration, including TG, is VL- and AIDS-driven prolonged inflammation leading to cachexia, promoting lipolysis and mobilization of TG and cholesterol esters from plasma and adipose tissues to hepatocytes. This profile also suggests a two-step process, which initially causes progressive depletion of fat stores and then reduces lipid recycling and availability for liver metabolism and synthesis of TC, TG, VLDL, and LDL [38] in a process that could be coined as fat metabolism exhaustion. Further investigations are required to confirm the finding of fat exhaustion in other protracted diseases with cachexia and inflammation.

An unexpected and intriguing discovery is the association of extremely low LDL with four late deaths of patients with relapsing VL and AIDS. This finding contradicts a recent review concluding that very low levels of LDL are not risky for the general population [39], although other authors found an increased risk of ischemic hemorrhagic stroke and death [40]. Moreover, a meta-analysis showed that mortality by sepsis was associated with lower TC, HDL, and LDL, albeit not with TG levels [41]. Hence, the idea that fat exhaustion led to the severe unavailability of lipids to synthesize vital lipid compounds for vitamin transport, cell membranes, the central nervous system, and steroid hormones is of interest [33]. However, the causal association of low LDL with death persists disputed, and the precise mechanisms of how low LDL would be related to death remain elusive.

The data showed that the lipid profile did not influence the action of amphotericin B on the relapsing course of VL in AIDS patients. First, the only causal relationship investigated through time, e.g., the amount of liposomal amphotericin accumulated, was not associated with any component of the lipid profile. Secondly, an experimental study with rabbits using liposomal amphotericin B [11] suggested that the higher the ingestion of cholesterol and the higher TC and LDL, the less toxic liposomal amphotericin B was, and a minor concentration of amphotericin B in the spleen was reached. Hence, as we found very low values of all lipids investigated, the hypothesis of high lipids reducing the effectiveness of amphotericin B can be ruled out. Unfortunately, amphotericin B concentration was not evaluated here. Therefore, it may be concluded that low CD4+ and, later, low CD8+ cells and low lipids were the main host factors for relapses of VL in patients with HIV.

The main limitation of the study is the small sample size. Therefore, type 2 errors may have hidden associations, such as the role of CD8+ cells with the course of relapsing VL. The cross-sectional design constrained causal inferences such as the ones of the lymphocyte counts and plasma lipids with relapses. However, the associations with death were necessarily longitudinal observations in a retrospective cohort design, allowing sound causal inferences regarding low LDL with mortality. Finally, since the patients in the first VL episode may have been mixed with future relapsing cases, other associations may have been missed.

In conclusion, the study reveals that resistance to amphotericin B or lipid interference with its activity are not the reasons for relapsing VL in patients with AIDS. Lymphopenia due to low CD4+ added to by a subsequent fall of CD8+ may be the late immunological factor involved in relapses. The lipid profile of low TC, HDL, LDL, and TG may also be a marker of relapse, and lipid exhaustion with extremely low LDL may indicate a higher probability of death.

The control of *Leishmania* with combination therapy, as recommended by the World Health Organization, is an immediate target for clinical trials in this population of patients, such as with amphotericin B and miltefosine or pentamidine and paromomycin. Since the spleen is also a sanctuary of HIV, more appropriate antiretroviral therapy with drugs with higher human spleen availability, like emtricitabine or, eventually, new drugs such as cabotegravir, is worth trying. Reducing inflammation may prevent CD4+ and CD8′ falls with steroids, mesalamine, statins, and others is worthy of clinical trials. For raising CD4+ and CD8+, recombinant IL-7 and immune checkpoint inhibitors for relapsing VL might be conducted for patients with relapsing VL and AIDS. Finally, the association of a two-step chronic inflammation caused by the two diseases leading first to CD4+ depletion followed by CD8+ falls, progressing to the exhaustion of lipids, may be intimately linked and deserves further cohort studies with patients who developed AIDS and its coinfections, especially those with a relapsing course such as VL. Finally, an obvious trial is to evaluate the use of cholesterol-rich diets for those patients at risk of death with very low LDL.

## Figures and Tables

**Table 1:** EC_50_ ¹ values of promastigotes from isolates taken from individuals with visceral leishmaniasis coinfected with HIV and of promastigotes of a reference strain.

Patient ID	Mean EC_50_ ¹	Standard Error	No. of Experiments	No. of Previous VL Episodes
	(µM)	(µM)		
5567 ^2^	0.042	0.007	5	2
5768 ^2^	0.037	0.006	5	2
6905 ^2^	0.034	0.007	4	2
7053 ^2^	0.024	0.008	5	2
5609 ^3^	0.026	0.006	4	0
MHOM/BR/2005/NLC ^4^	0.051	0.005	3	-

^1^ Half-maximal effective concentration. ^2^ The promastigotes used in the experiment were isolated from patients who were in the second episode of visceral leishmaniasis. ^3^ The isolate used in the experiment was taken from a patient in the first episode of visceral leishmaniasis. ^4^ Reference isolate.

**Table 2:** Demographic and clinical characteristics of individuals with visceral leishmaniasis infected with HIV.

Characteristics	First Episode n (%)	Relapsing n (%)	*p*-Value
Sex			
Male	6 (100.0)	20 (87.0)	
Female	0 (0.0)	3 (13.0)	0.350
Age group			
Up to 40 years old	4 (66.7)	8 (34.8)	
More than 40 years	2 (33.3)	15 (65.2)	0.158
Symptoms and signs			
Weight loss	6 (100.0)	17 (73.4)	0.160
Pallor	5 (83.3)	17 (73.4)	0.631
Fever	6 (100.0)	13 (56.5)	0.046
Asthenia/weakness	4 (66.7)	14 (60.9)	0.794
Splenomegaly	3 (50.0)	13 (56.5)	0.775
Hepatomegaly	2 (33.3)	9 (39.1)	0.794
Jaundice	2 (33.3)	8 (34.8)	0.947
HAART *	6/6 (100.0)	15/23 (0.65)	0.43
Death	0 (0.0)	4 (17.4)	0.55

* Highly active antiretroviral therapy.

**Table 3:** Laboratory data of individuals with visceral leishmaniasis infected with HIV, according to the course of VL (relapsing and first episode).

Laboratory Data	Course of VL				*p*-Value ^c^
	First Episode (n = 6)		Relapsing (n = 23)		
	n ^a^	Mean (95% CI ^b^)	n ^a^	Mean (95% CI)	
Hemoglobin (g/dL)	5	8.0 (3.6; 12.5)	19	7.8 (7.2; 8.4)	0.522
Leukocytes (per mm^3^)	5	4.643 (1060; 4; 8.223)	21	2.397 (1713; 3.083)	0.013
Neutrophils (cells/mm^3^)	5	2410 (−272; 5092.9)	19	1409 (889; 1930)	0.076
Lymphocytes (cells/mm^3^)	5	1654 (−88; 3396)	19	660 (457; 864)	0.005
Platelets (number/mm^3^)	6	151,952 (151,952;184,813)	21	190,217 (89.298; 291,135)	0.297
Albumin (g/dL)	1	1.8	10	2.7 (1.9; 3.6)	-
Globulin (g/dL)	1	9.9	9	6.0 (4.8;7.2)	-
CD4+ (cells/mm^3^)	5	152 (−32; 332)	15	142 (89; 196)	0.877
CD8+ (cells/mm^3^)	5	955 (−197.4; 2106.55)	14	606 (423; 789)	0.222

^a^ Number of patients with the information available; ^b^ 95% confidence interval; ^c^ Student’s *t*-test.

**Table 4:** Association between the lipid profile and the clinical outcome of individuals with visceral leishmaniasis coinfected with HIV according to survival and death.

Lipid Profile	Outcome		*p-*Value
	Survival (n ^a^ = 25) Mean (95% CI ^b^)	Death (n = 4) Mean (95% CI)	
TC ^c^ (mg/dL)	68.3 (56.1; 80.5)	60.7 (20.8; 100.7)	0.633
LDL ^d^ (mg/dL)	20.4 (14.2; 26.7)	3.6 (−3.8; 10.9)	0.038
HDL ^e^ (mg/dL)	32.7 (25.3; 40.2)	37.3 (8.6; 65.9)	0.644
VLDL ^f^ (mg/dL)	15.2 (12.7; 17.6)	20.0 (5.7; 34.2)	0.170
TG ^g^ (mg/dL)	75.8 (63.7; 88.0)	99.8 (28.4; 171.1)	0.170

^a^ Number of individuals. ^b^ 95% confidence interval. ^c^ Total cholesterol. ^d^ Low-density lipoprotein. ^e^ High-density lipoprotein. ^f^ Very low-density lipoprotein. ^g^ Triglycerides.

## Data Availability

Data are contained within the article are available by e-mail to CHNC.

## Supplementary Materials

The following supporting information can be downloaded at: https://www.mdpi.com/article/10.3390/pathogens13060450/s1, Table S1: Comparison of the lipid profile of 29 individuals with relapsing and non-relapsing visceral leishmaniasis infected with HIV with the desirable values.
