# Supplementary material for: Low Plasma Lipids Are Associated with Relapsing and Lethal Visceral Leishmaniasis in HIV-Infected Patients
Source: Pathogens. 2024 May 25;13(6):450. doi: 10.3390/pathogens13060450 (PMC11206293; doi:10.3390/pathogens13060450)
Supplement: Supplementary file 1 [file pathogens-13-00450-s001.zip › pathogens-3010950-supplementary.pdf]

**Supplemental table**. Comparison of the lipid profile of 29 individuals with relapsing and non-relapsing visceral leishmaniasis infected with HIV with the desirable values.

| Lipid profile | Mean (95% CI[a]) | Median (IQ[b]) | Desirable values (mg/dL) |
|---|---|---|---|
| Total cholesterol (mg/dL) | 67.3 (56.4;78.2) | 66.0 (59.5;75.5) | < 190 mg/dL |
| LDL[c] (mg/dL) | 18.1 (12.3;23.9) | 15.8 (6.4; 22.8) | < 130 mg/dL |
| HDL[d] (mg/dL) | 33.3 (26.6;32.9) | 35.0 (17.0;41.5) | > 40 mg/dL |
| VLDL[e] (mg/dL) | 15.8 (13.4;18.3) | 15.2 (10.8; 19.6) | < 45 mg/dL |
| Triglycerides (mg/dL) | 79.1 (66.9;91.3) | 76.0 (54.0;102.5) | < 175 mg/dL |

[a] 95% confidence interval. [b] Interquartile range. [c] Low-density lipoprotein. [d] High-density lipoprotein. [e] Very low-density lipoprotein.
